# Time trends of vitamin D concentrations in northern Sweden between 1986 and 2014: a population-based cross-sectional study

**DOI:** 10.1007/s00394-019-02142-x

**Published:** 2019-11-21

**Authors:** Eva Summerhays, Mats Eliasson, Robert Lundqvist, Stefan Söderberg, Tanja Zeller, Viktor Oskarsson

**Affiliations:** 1 Sunderby Research Unit, Department of Public Health and Clinical Medicine, Umeå University, 901 87 Umeå, Sweden; 2 Research and Innovation Unit, Norrbotten County Council, Luleå, Sweden; 3 Unit of Medicine, Department of Public Health and Clinical Medicine, Umeå University, Umeå, Sweden; 4 Department of General and Interventional Cardiology, University Heart Center Hamburg, Hamburg, Germany; 5 German Center for Cardiovascular Research, Partner Site Hamburg/Kiel/Luebeck, Hamburg, Germany

**Keywords:** Vitamin D, Trend, Cross-sectional study, MONICA, Sweden

## Abstract

**Purpose:**

Vitamin D, produced through cutaneous photosynthesis or ingested via foods or supplements, has generated considerable research interest due to its potential health effects. However, epidemiological data on the time trends of vitamin D status are sparse, especially from northern Europe. We examined the time trend of vitamin D concentrations in northern Sweden between 1986 and 2014.

**Methods:**

We used data on 11,129 men and women (aged 25–74 years) from seven population-based surveys (the Northern Sweden MONICA study), recruited between 1986 and 2014. Serum vitamin D (25-hydroxyvitamin D) status was measured using a one-step immunoassay (Abbott Architect). Multivariable linear regression models, adjusted for age, sex, and a number of other variables, were used to estimate the time trend of vitamin D concentrations.

**Results:**

The mean value of vitamin D in the entire study population was 19.9 ng/mL [standard deviation (SD) 7.9], with lower values in men (19.4 ng/mL; SD 7.5) than in women (20.5 ng/mL; SD 8.2). Using the survey in 1986 as reference category, the multivariable-adjusted mean difference [95% confidence interval (CI)] in ng/mL was 2.7 (2.2, 3.3) in 1990, 3.2 (2.7, 3.7) in 1994, 1.6 (1.0, 2.1) in 1999, − 2.0 (− 2.5, − 1.4) in 2004, 1.0 (0.4, 1.5) in 2009, and 3.1 (2.5, 3.6) in 2014.

**Conclusion:**

In this large cross-sectional study, we observed no clear upward or downward trend of vitamin D concentrations in northern Sweden between 1986 and 2014.

**Electronic supplementary material:**

The online version of this article (10.1007/s00394-019-02142-x) contains supplementary material, which is available to authorized users.

## Introduction

Vitamin D, which is produced by cutaneous synthesis from sunlight exposure or ingested via foods or supplements, refers to a group of fat-soluble compounds that are essential for adequate mineral balance [1]. Subsequently, vitamin D has a role in musculoskeletal health, with low concentrations of vitamin D being associated with muscular function, osteoporosis, and fractures [2, 3]. During the last decades, there has also been mounting evidence from observational studies that vitamin D might be associated with several non-skeletal diseases (e.g., cancer [4], autoimmune disease [5], cardiovascular disease [6], fatigue [7], and depression [8]) as well as mortality [9]. It is, however, unclear whether these are causal associations, exemplified by the non-effect of vitamin D supplementation on musculoskeletal health in randomized controlled trials (as meta-analyzed by Bolland et al. [10]).

The northern European population is considered at risk for vitamin D insufficiency, because of the undetectable cutaneous synthesis of vitamin D at high latitudes during the winter [11], leaving inhabitants more dependent on dietary sources of vitamin D. A number of countries have, therefore, introduced a vitamin D food-fortification policy [including Sweden, where it was recently expanded [12] (see Online Resource 1 for details)]. Previous studies from northern Europe have mainly focused on the prevalence of vitamin D insufficiency (< 20 ng/mL), which has ranged from 4 to 77% (as reviewed by Ramnemark et al. [13]), and only two larger studies have examined the time trend of vitamin D status. One Norwegian study reported a slight increase in mean vitamin D values between 1994 and 2008 (from 21.5 to 22.2 ng/mL) [14], while a Finnish study reported a larger increase in mean vitamin D values between 2000 and 2011 (from 19.2 to 26.0 ng/mL) [15].

The aim of this study was to examine whether the vitamin D concentrations in northern Sweden had increased between 1986 and 2014, which we expected due to the ongoing debate over vitamin D and the altered food-fortification policies, using data from seven population-based surveys.

## Subjects and methods

### Study population

Data were obtained from the northern Sweden component of the MONICA study (MONItoring of trends and determinants in CArdiovascular disease). In total, between 1986 and 2014, seven population-based surveys were conducted in Norrbotten and Västerbotten counties. In these counties, the two major population centers are Luleå (at latitude 65.7° N) and Umeå (at latitude 63.9° N). All surveys were conducted during the same months (between January and April) and each individual filled in a questionnaire on lifestyle habits, underwent a clinical examination, and had blood samples drawn. Individuals were randomly selected from population registers and stratified for age (25–64 years in 1986 and 1990; 25–74 years in 1994, 1999, 2004, 2009, and 2014) and sex. The seven samples were selected independently of each other and have all had good participation rates, with estimates ranging from 81% in 1986 to 63% in 2014. Details on sampling and selection, as well as on non-participation data, have been published elsewhere [16, 17].

The Northern Sweden MONICA study has been covered by multiple ethical approvals from the Regional Ethical Committee at Umeå University (Umeå, Sweden) from its initiation up until 2013. The recommendations of the Strengthening the Reporting of Observational Studies in Epidemiology (STROBE) initiative were followed whenever applicable [18].

### Assessment of vitamin D concentrations

Blood samples were drawn after at least a 4-h fast and stored at − 80 °C. Serum 25-hydroxyvitamin D, which is the circulating biomarker of vitamin D status, was analyzed between 2016 and 2018 at the University Medical Center Hamburg-Eppendorf (Hamburg, Germany) within the frame of the European BiomarCaRE project (Biomarkers for Cardiovascular Risk Assessment in Europe) [19] and measured using a one-step immunoassay on the Abbott Architect i2000 (Abbott Laboratories, Abbott Park, IL, USA) [20]. The valid measurement range was 8–160 ng/mL. The intra- and inter-assay coefficient of variation (based on different concentrations of control samples) was 1.10–2.99% and 4.31–9.11%, respectively. (Hereinafter, for simplicity, serum 25-hydroxyvitamin D will only be referred to as vitamin D.)

Vitamin D concentrations in the survey in 2009 have previously been measured using high-performance liquid chromatography (HPLC) [13], which is considered the golden standard method for vitamin D analyses [21]. We, therefore, compared the Abbott Architect data with the HPLC data. The correlation between measurement methods was high in terms of rank [Spearman’s coefficient (*r*) = 0.85], irrespective of sex (*r *= 0.86) and age group (*r *= 0.82–0.91). However, as shown in Online Resource 2, the concentrations of vitamin D were on average 8.4 ng/mL [standard deviation (SD) 4.8] lower in the Abbott Architect data, with seemingly larger between-method differences for higher values of vitamin D. The mean difference in vitamin D concentrations between the measurement methods was fairly consistent in men and women as well as in different age groups (Online Resource 3).

### Assessment of other variables

On each questionnaire, participants were asked to report their country of birth, civil status, educational level, cigarette use, snus use (i.e., moist oral snuff), and physical activity. Height and weight were measured during the clinical examination, and body mass index (BMI) was calculated as weight (kg) divided by height squared (m^2^).

### Statistical analysis

Of the 12,130 participants who were eligible for the current study, we excluded 33 with missing data on survey year and 968 with missing or invalid data on vitamin D concentrations.

Means and percentages of demographic, behavioral, and health characteristics (including vitamin D concentrations) in the seven surveys were standardized to the sex and age (< 35, 35–44, 45–54, ≥ 55 years) distribution of the entire Swedish population in 2000.

Time trends of vitamin D concentrations according to survey year [1986 (ref), 1990, 1994, 1999, 2004, 2009, and 2014] were analyzed by using a linear regression model, adjusted for sex and age (continuous, years). Since there was evidence of a non-linear association between vitamin D concentrations and age, we modeled age by using 4-knot restricted cubic splines (at the 5th, 35th, 65th, and 95th percentile of the age distribution) [22]. To further examine the distribution of vitamin D concentrations, we used quantile regression models to calculate age- and sex-adjusted percentile values for each survey year.

Multivariable linear regression models were adjusted for month of sampling (January or February, March or April), country of birth (Sweden, other), civil status (married or cohabiting, other), educational level (university, non-university), smoking status (current, non-current), and BMI (< 25, 25–29, ≥ 30 kg/m^2^). Additional adjustment for county of residence (Norrbotten, Västerbotten) and snus status (current, non-current) did not change the results (data not shown). The missing-indicator method was used to handle missing covariate data in the multivariable model (overall fraction of 2.3%); however, results obtained from a complete case analysis were identical (data not shown).

Separate linear regression analyses of (1) men and women, (2) different age groups (< 35, 35–44, 45–54, ≥ 55 years), and (3) month of sampling (January, February, March, April) were performed as sensitivity analyses. Since the data on vitamin D concentrations were somewhat right-skewed, we performed additional sensitivity analyses by excluding participants with vitamin D concentrations greater than 75, 60, and 50 ng/mL, respectively. In a final sensitivity analysis, the multivariable model was restricted to the surveys in 1990–2009 and further adjusted for physical activity (almost none, light-effort ≥ 1 h/week, high-effort ≥ 1 h/week), since this variable was only measured in a consistent way between 1990 and 2009.

Statistical significance was set at a two-sided *P* value less than 0.05. Analyses were performed using Stata version 14 (StataCorp LP, College Station, TX, USA).

## Results

A total of 11,129 participants (5459 men and 5670 women), recruited from the seven surveys conducted between 1986 and 2014, were included in the analysis (Online Resource 4). Characteristics of the study population by survey year are shown in Table 1. Compared with participants in the surveys in 1986 and 1990, participants in later surveys were more likely to be older, married or cohabiting, well educated, physically active, and to have a BMI of 30 kg/m^2^ or more. These participants were also less likely to smoke cigarettes. The percentage of participants sampled in January or February varied greatly in the surveys in 1986–1999 (from 37.0 to 73.5%), whereas the same percentage was stable in the surveys in 2004–2014 (from 56.5 to 58.8%).

**Table 1:** Characteristics of the study population (*n* = 11,129) by survey year, 1986–2014

	Survey year						
Characteristics^a,b^	1986	1990	1994	1999	2004	2009	2014
Participants (*n*)	1571	1393	1836	1670	1658	1554	1447
Mean age (years)^c^	45.2	45.2	49.9	51.0	51.4	51.2	51.8
Male sex (%)	50.8	48.4	48.9	49.0	48.6	49.8	47.8
Sampled in January or February (%)	73.5	57.5	64.6	37.0	58.8	58.2	56.5
Born in Sweden (%)	94.7	93.6	94.1	93.3	94.1	93.8	91.8
Married or cohabiting (%)	80.7	80.1	76.0	71.8	76.3	71.7	65.8
University education (%)	11.5	16.3	19.3	23.1	29.7	32.5	34.0
Current smoker (%)^d^	31.0	29.2	25.4	17.7	16.0	14.7	12.6
High-effort physical activity ≥ 1 h/week (%)^e^	–	32.2	31.3	33.6	39.7	41.4	–
Body mass index ≥ 30 kg/m^2^ (%)	10.2	9.5	12.7	15.0	18.0	19.2	18.6

^a^Values (except age, sex, month of sampling, and country of birth) were standardized to the sex and age (< 35, 35–44, 45–54, ≥ 55 years) distribution of the entire Swedish population in 2000

^b^Values were calculated for participants with complete data. Data were missing on the month of sampling for three participants, on the country of birth for 57 participants, on civil status for 51 participants, on educational level for 147 participants, on smoking status for 40 participants, on physical activity for 171 participants, and on body mass index for 45 participants

^c^The age structure of included participants was 25–64 years in 1986–1990 and 25–74 years in 1994–2014

^d^Includes occasional (< 1 cigarette/day) smokers

^e^The question on leisure time physical activity was vastly different in the surveys in 1986 and 2014 compared to the other surveys; therefore, it was not possible to create a joint variable across all surveys

The mean value of vitamin D in the entire study population was 19.9 ng/mL (SD 7.9), with lower values in men (19.4 ng/mL; SD 7.5) than in women (20.5 ng/mL; SD 8.2) [*P* value (*t* test) < 0.001]. Participants aged ≥ 55 years had a higher mean value (20.7 ng/mL; SD 8.1) than those aged 45–54 years (19.6 ng/mL; SD 7.4), 35–44 years (19.2 ng/mL; SD 7.5), and ≤ 34 years (19.7 ng/mL; SD 8.2) [*P* value (analysis of variance) < 0.001].

As shown in Table 2, there was no clear upward or downward trend of vitamin D concentrations between 1986 and 2014: the age- and sex-standardized mean value across the seven surveys was 18.4, 21.1, 22.0, 19.6, 17.0, 19.4, and 21.1 ng/mL, respectively. The mean value in 2004 (17.0 ng/mL) was especially noteworthy, as it was substantially lower than in all other surveys. In a multivariable linear regression model, which used the survey in 1986 as reference category, the mean difference [95% confidence interval (CI)] in ng/mL was 2.7 (2.2, 3.3) in 1990, 3.2 (2.7, 3.7) in 1994, 1.6 (1.0, 2.0) in 1999, − 2.0 (− 2.5, − 1.4) in 2004, 1.0 (0.4, 1.5) in 2009, and 3.1 (2.5, 3.6) in 2014. The variable with the highest impact on the regression estimates was month of sampling; the other variables had only little to marginal impact.

**Table 2:** Vitamin D concentrations in the study population by survey year, 1986–2014

	Survey year						
Vitamin D concentration (ng/mL)	1986	1990	1994	1999	2004	2009	2014
Mean value							
Crude	18.6	21.2	22.0	19.8	16.7	19.7	21.7
Age and sex standardized^a^	18.4	21.1	22.0	19.6	17.0	19.4	21.1
Mean difference							
Age and sex adjusted (95% CI)	Ref.	2.5 (1.9, 3.0)	3.0 (2.5, 3.6)	0.9 (0.4, 1.4)	− 2.3 (− 2.8, − 1.7)	0.7 (0.1, 1.2)	2.7 (2.2, 3.3)
Multivariable adjusted (95% CI)^b^	Ref.	2.7 (2.2, 3.3)	3.2 (2.7, 3.7)	1.6 (1.0, 2.1)	− 2.0 (− 2.5, − 1.4)	1.0 (0.4, 1.5)	3.1 (2.5, 3.6)

*CI* confidence interval

^a^Standardized to the sex and age (< 35, 35–44, 45–54, ≥ 55 years) distribution of the entire Swedish population in 2000

^b^Estimated from a linear regression model adjusted for sex, age (continuous using 4-knot restricted cubic splines, years), month of sampling (January/February, March/April), country of birth (Sweden, other), civil status (married/cohabiting, other), educational level (university, non-university), smoking status (current, non-current), and body mass index (< 25, 25–29, ≥ 30 kg/m^2^)

The shape of the association between vitamin D concentrations and survey year was consistent across sex and age group (Table 3) as well as month of sampling (data not shown). The results were also similar in the sensitivity analyses in which we excluded participants with vitamin D values greater than 50 (*n* = 62), 60 (*n* = 19), and 75 ng/mL (*n* = 5). As an example, when those with values greater than 50 ng/mL were excluded, and once again using the survey in 1986 as reference category, the multivariable-adjusted mean difference (95% CI) in ng/mL was 2.7 (2.2, 3.2) in 1990, 3.1 (2.6, 3.6) in 1994, 1.4 (0.9, 1.9) in 1999, − 2.0 (− 2.5, − 1.5) in 2004, 0.7 (0.2, 1.2) in 2009, and 2.6 (2.1, 3.1) in 2014. Finally, when restricting the analysis to the surveys in 1990–2009, to further adjust the multivariable model for physical activity, the results did not substantially change [multivariable-adjusted mean difference (95% CI) in ng/mL: 0.6 (0.1, 1.1) in 1994, − 1.1 (− 1.6, − 0.5) in 1999, − 4.7 (− 5.2, − 4.1) in 2004, and − 1.8 (− 2.3, − 1.2) in 2009; compared to the survey in 1990].

**Table 3:** Vitamin D concentrations in the study population by survey year and according to sex and age, 1986–2014

	Survey year						
Vitamin D concentration (ng/mL)	1986	1990	1994	1999	2004	2009	2014
Men (*n* = 5459)							
Standardized mean value^a^	18.1	20.5	20.8	18.9	16.6	18.6	19.5
Adjusted mean difference (95% CI)^b^	Ref.	2.4 (1.7, 3.2)	2.5 (1.8, 3.2)	1.2 (0.5, 2.0)	− 2.2 (− 2.9, − 1.5)	0.6 (− 0.1, 1.3)	1.4 (0.7, 2.2)
Women (*n* = 5670)							
Standardized mean value^a^	18.7	21.7	23.2	20.4	17.4	20.1	22.7
Adjusted mean difference (95% CI)^b^	Ref.	3.0 (2.2, 3.8)	3.9 (3.2, 4.7)	2.0 (1.2, 2.8)	− 1.7 (− 2.5, − 0.9)	1.3 (0.5, 2.1)	4.7 (3.8, 5.5)
Age < 35 years (*n* = 1959)							
Standardized mean value^a^	18.1	20.9	22.3	19.4	17.7	19.0	20.2
Adjusted mean difference (95% CI)^b^	Ref.	3.2 (2.0, 4.4)	4.5 (3.4, 5.7)	2.0 (0.7, 3.2)	0.0 (− 1.3, 1.3)	1.3 (− 0.1, 2.6)	2.5 (1.1, 3.9)
Age 35–44 years (*n* = 2303)							
Standardized mean value^a^	18.9	21.1	20.8	18.6	15.8	18.2	20.2
Adjusted mean difference (95% CI)^b^	Ref.	2.3 (1.3, 3.4)	2.0 (1.0, 3.1)	0.5 − 0.7, 1.6)	− 2.7 (− 3.8, − 1.6)	− 0.4 (− 1.5, 0.7)	1.7 (0.5, 2.9)
Age 45–54 years *(n* = 2481)							
Standardized mean value^a^	19.3	21.0	21.4	19.4	15.9	18.8	20.6
Adjusted mean difference (95% CI)^b^	Ref.	2.1 (1.1, 3.1)	2.3 (1.3, 3.3)	0.7 (− 0.3, 1.8)	− 3.1 (− 4.2, − 2.1)	− 0.1 (− 1.2, 1.0)	1.8 (0.7, 2.9)
Age ≥ 55 years (*n* = 4386)							
Standardized mean value^a^	18.2	21.6	22.6	20.7	17.0	20.9	23.3
Adjusted mean difference (95% CI)^b^	Ref.	3.4 (2.3, 4.5)	4.6 (3.6, 5.5)	3.2 (2.2, 4.1)	− 0.9 (− 1.9, 0.0)	2.9 (1.9, 3.8)	5.3 (4.3, 6.2)

*CI* confidence interval

^a^Standardized to the age (< 35, 35–44, 45–54, ≥ 55 years) and sex distribution, respectively, of the entire Swedish population in 2000

^b^Estimated from a linear regression model adjusted for sex, age (continuous using 4-knot restricted cubic splines, years), month of sampling (January/February, March/April), country of birth (Sweden, other), civil status (married/cohabiting, other), educational level (university, non-university), smoking status (current, non-current), and body mass index (< 25, 25–29, ≥ 30 kg/m^2^)

**Fig. 1:**
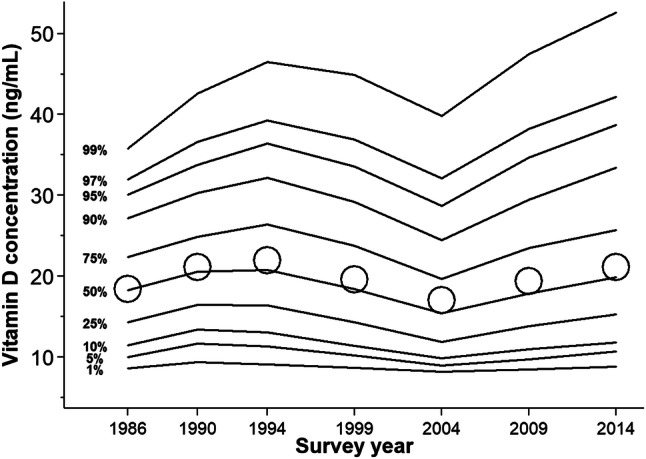
Percentile and mean distribution of vitamin D concentrations in the study population by survey year, 1986–2014. The solid lines represent percentile values, which were estimated from a quantile regression model adjusted for sex and age (continuous, years). The hollow circles represent mean values (added for comparison), which were standardized to the sex and age (< 35, 35–44, 45–54, ≥ 55 years) distribution of the entire Swedish population in 2000

Overall, the association between different percentile values of vitamin D concentrations and survey year was similar to the association based on mean values (Fig. 1). The later surveys (2004, 2009, and 2014) had the most right-skewed data distributions, especially that in 2014, for which the 97th to 99th percentile values were markedly higher (42.2 and 52.5 ng/mL, respectively) than in the other surveys (32.0 to 39.3 ng/mL and 35.8 to 46.5 ng/mL, respectively). The 1st and 5th percentile values ranged between 8.2 (2004) to 9.4 (1990) ng/mL and 9.0 (2004) to 11.7 (1990) ng/mL, respectively.

## Discussion

In this large cross-sectional study, based on data from seven population-based surveys, we observed no clear upward or downward trend of vitamin D concentrations in northern Sweden between 1986 and 2014. Compared to 1986, the mean vitamin D value was largely increased in 1990, 1994, and 2014, marginally increased in 1999 and 2009, and decreased in 2004.

In the ongoing debate as to whether low population concentrations of vitamin D are of public health importance, the northern European population is of special interest due to its high-latitude habitat, which, during the winter, leads to insufficient exposure to the amount and type of sunlight that is required for cutaneous synthesis of vitamin D [11]. To counter this, several northern European countries have long had vitamin D food-fortification policies. In Sweden, the vitamin D food-fortification policy was recently expanded [12]. As shown in Online Resource 1, the current policy includes more food items and demands a higher quantity of vitamin D in each food item.

Our findings are in somewhat contrast to those in previous large studies from northern Europe. In a Norwegian longitudinal study of 2668 non-smoking individuals, there was a small increase in mean vitamin D concentrations between 1994 and 2008 (from 21.5 to 22.2 ng/mL) [14]. A larger increase in mean vitamin D concentrations was observed in a Finnish cross-sectional study of 10,185 individuals: from 19.2 ng/mL in 2000 to 26.0 ng/mL in 2011 [15]. Compared to our study, which had seven measurement points over a follow-up period of 28 years, these studies only had two measurement points—an important difference that hinders between-study comparisons. If we, for example, only had had data between 1986 and 1994 or between 2004 and 2014, we would also have concluded that the mean vitamin D concentrations in northern Sweden had increased over time (from 18.4 to 22.0 ng/mL and from 17.0 to 21.1 ng/mL, respectively). Thus, as highlighted by our study, it is important to have multiple measurement points when examining a variable like vitamin D, which will vary with the amount of sunlight in a given year and with individual sunlight exposure. It should also be noted that there are other studies, from countries outside of northern Europe, which have shown both increased (e.g., Canada [23], the US [24, 25], Iran [26], and Ireland [27]) and decreased (e.g., the USA [28, 29], Greenland [30], and South Korea [31]) vitamin D concentrations over time.

It is, from a biological standpoint, hard to understand the observed fluctuation of vitamin D status in our study, especially the very low value in 2004. However, despite the adjustment for a number of variables in the linear regression model, we cannot exclude residual confounding (e.g., due to misreporting of smoking status, educational level, and physical activity) and—more importantly—unmeasured confounding as potential explanations. Firstly, we had no data on sunlight exposure; that is, the most important determinant of vitamin D status. Using official statistics from the Swedish Meteorological and Hydrological Institute [32], we tried to examine whether the hours of sunlight had differed between the survey years. However, the total hours of sunlight during the preceding year, as well as during the sampling period from January to April, was not substantially different in 2004 compared to 2009 and 2014. We also lacked data on sun holidays, which, in theory, could explain some (but hardly all) of the observed variance. Secondly, in addition to the fact that we did not account for differences in food and beverage consumption, the food-fortification policy in Sweden changed during the study period. In 2007, after regulations in the European Parliament and the European Council, fortification with certain enrichments became compulsory (before which it was voluntary for non-organic items and prohibited for organic items), vitamin D being one of them [33, 34]. Thus, it is possible that the increase in mean vitamin D concentrations in 2009 and 2014 compared to 2004 was due to the instatement of mandatory vitamin D food fortification. One can also speculate if the voluntary vitamin D food fortification prior to 2007 was different between the survey years. Finally, we only had data on vitamin D supplementation in the survey in 2009, leaving the possibility of unmeasured confounding. Around 14% of the participants in 2009 used vitamin D supplements; and they had an almost 3 ng/mL higher mean vitamin D value than non-users [13], indicating a strong potential for confounding if the use of vitamin D supplements varied by survey year. Likewise, we had no data on physician-prescribed vitamin D treatment. In 2011, although not without controversy, the Endocrine Society’s clinical practice guideline suggested that at-risk individuals of vitamin D insufficiency (listed, among others, are pregnant women, obese adults, and older people with a history of falls) should be screened and, if concentrations are less than 20 ng/mL, treated to achieve concentrations of at least 30 ng/mL [35]. While we are not aware of such widespread vitamin D screening having been implemented in northern Sweden, it is possible that screening (and subsequent treatment) increased to some extent post-2011, which, in turn, might have contributed to the rather high 97th to 99th percentile values in the survey in 2014.

As an additional explanation to the very low value of vitamin D in 2004, we have given considerable thought on the possibility of some sort of pre-analytical or laboratory error. However, the frozen blood samples from each survey were handled in an identical and standardized way before shipment for analysis (Stefan Söderberg, the Northern Sweden MONICA study, personal communication, 2019), not to mention that blood samples of vitamin D are robust to handling [36] and storage duration [37] and can withstand multiple freeze–thaw cycles [38]. There was also no evidence of inconsistencies or errors in the laboratory procedures of the blood samples in 2004 (or in the other surveys) at retrospective review. Other biomarkers from the Northern Sweden MONICA study, such as troponin and *N*-terminal pro-B-type natriuretic peptide, have not had markedly lower values in 2004 compared to the other survey years (Tanja Zeller, the European BiomarCaRE project, unpublished data, 2019).

Another important limitation of the study must be mentioned, which is the fact that the vitamin D status was measured using a one-step immunoassay (Abbott Architect) instead of an HPLC: that is, the method considered to be the golden standard for vitamin D analyses [21]. In our own validation, using the survey in 2009, which had previously been analyzed with HPLC, the concentrations of vitamin D were on average 8.4 ng/mL lower in the Abbott Architect data compared to the HPLC data. Assuming the same level of underestimation by the Abbott Architect in each survey year, the mean value of vitamin D in the seven surveys would have been 26.8, 29.5, 30.4, 28.1, 25.4, 27.8, and 29.5 ng/mL, respectively, if the analysis had been conducted with HPLC. As a consequence, on an absolute scale, the observed mean values of vitamin D should be interpreted with caution [e.g., the percentage of participants having adequate vitamin D concentrations (≥ 20 ng/mL, as specified by the US Institute of Medicine [39]) in the survey in 2009 was 83% using the HPLC data, but only 40% using the Abbott Architect data]. However, the methods had a good correlation in terms of rank and we have no reason to suspect that the measurement error should vary by survey year, especially since all samples were analyzed by the same laboratory and at the same time; therefore, the relative differences in mean values of vitamin D should be valid.

Strengths of this study included the large sample size, as well as the fairly high response rate in each survey, which increases the study’s probability of being an accurate representation of the general population in northern Sweden. In addition, the study covered a time period of 28 years, with a total of seven measurement points, allowing us to perform one of the most detailed analyses on the time trend of vitamin D status to date.

In summary, in this population-based cross-sectional study, we observed no clear upward or downward trend of vitamin D concentrations in northern Sweden between 1986 and 2014. Whether this finding is of public health importance, and whether it gives justification to the recently expanded vitamin D food-fortification policy in Sweden, is up for debate and calls for further research.

## Electronic supplementary material

Below is the link to the electronic supplementary material.

Supplementary material 1 (DOCX 45 kb)

## References

[1] Hossein-nezhad A, Holick MF (2013). Vitamin D for health: a global perspective. Mayo Clin Proc.

[2] Holick MF (2004). Vitamin D: importance in the prevention of cancers, type 1 diabetes, heart disease, and osteoporosis. Am J Clin Nutr.

[3] Lai JK, Lucas RM, Clements MS, Roddam AW, Banks E (2010). Hip fracture risk in relation to vitamin D supplementation and serum 25-hydroxyvitamin D levels: a systematic review and meta-analysis of randomised controlled trials and observational studies. BMC Public Health.

[4] Mondul AM, Weinstein SJ, Layne TM, Albanes D (2017). Vitamin D and cancer risk and mortality: state of the science, gaps, and challenges. Epidemiol Rev.

[5] Cantorna MT, Mahon BD (2004). Mounting evidence for vitamin D as an environmental factor affecting autoimmune disease prevalence. Exp Biol Med (Maywood).

[6] Kendrick J, Targher G, Smits G, Chonchol M (2009). 25-Hydroxyvitamin D deficiency is independently associated with cardiovascular disease in the third national health and nutrition examination survey. Artherosclerosis.

[7] Havdahl A, Mitchell R, Paternoster L, Davey Smith G (2019). Investigating causality in the association between vitamin D status and self-reported tiredness. Sci Rep.

[8] Lee DM, Tajar A, O’Neill TW (2011). Lower vitamin D levels are associated with depression among community-dwelling European men. J Psychopharmacol.

[9] Schöttker B, Jorde R, Peasey A (2014). Vitamin D and mortality: meta-analysis of individual participant data from a large consortium of cohort studies from Europe and the United States. BMJ.

[10] Bolland MJ, Grey A, Avenell A (2018). Effects of vitamin D supplementation on musculoskeletal health: a systematic review, meta-analysis, and trial sequential analysis. Lancet Diabetes Endocrinol.

[11] Webb AR, Kline L, Holick MF (1988). Influence of season and latitude on the cutaneous synthesis of vitamin D3: exposure to winter sunlight in Boston and Edmonton will not promote vitamin D3 synthesis in human skin. J Clin Endocrinol Metab.

[12] Livsmedelsverket (National Food Agency, Sweden) (2018) LIVSFS 2018:5 Livsmedelsverkets föreskrifter om berikning av vissa livsmedel (The National Food Agency’s regulations on the fortification of certain foods). www.livsmedelsverket.se/om-oss/lagstiftning1/gallande-lagstiftning/livsfs-20185. Accessed 25 Sept 2018

[13] Ramnemark A, Norberg M, Pettersson-Kymmer U, Eliasson M (2015). Adequate vitamin D levels in a Swedish population living above latitude 63°N: the 2009 Northern Sweden MONICA study. Int J Circumpolar Health.

[14] Jorde R, Sneve M, Hutchinson M, Emaus N, Figenschau Y, Grimnes G (2010). Tracking of serum 25-hydroxyvitamin D levels during 14 years in a population-based study and during 12 months in an intervention study. Am J Epidemiol.

[15] Jääskeläinen T, Itkonen ST, Lundqvist A (2017). The positive impact of general vitamin D food fortification policy on vitamin D status in a representative adult Finnish population: evidence from an 11-y follow-up based on standardized 25-hydroxyvitamin D data. Am J Clin Nutr.

[16] Stegmayr B, Lundberg V, Asplund K (2003). The events registration and survey procedures in the Northern Sweden MONICA Project. Scand J Public Health.

[17] Eriksson M, Holmgren L, Janlert U, Jansson JH, Lundblad D, Stegmayr B, Söderberg S, Eliasson M (2011). Large improvements in major cardiovascular risk factors in the population of northern Sweden: the MONICA study 1986-2009. J Intern Med.

[18] von Elm E, Altman DG, Egger M, Pocock SJ, Gøtzsche PC, Vandenbroucke JP (2007). Strengthening the reporting of observational studies in epidemiology (STROBE) statement: guidelines for reporting observational studies. BMJ.

[19] Zeller T, Hughes M, Tuovinen T (2014). BiomarCaRE: rationale and design of the European BiomarCaRE project including 300,000 participants from 13 European countries. Eur J Epidemiol.

[20] Cavalier E, Lukas P, Bekaert AC, Carlisi A, Le Goff C, Delanaye P, Souberbielle JC (2017). Analytical and clinical validation of the new Abbot Architect 25(OH)D assay: fit for purpose?. Clin Chem Lab Med.

[21] Snellman G, Melhus H, Gedeborg R, Byberg L, Berglund L, Wernroth L, Michaëlsson K (2010). Determining vitamin D status: a comparison between commercially available assays. PLoS ONE.

[22] Orsini N, Greenland S (2011). A procedure to tabulate and plot results after flexible modeling of a quantitative covariate. Stata J.

[23] Berger C, Greene-Finestone LS, Langsetmo L (2012). Temporal trends and determinants of longitudinal change in 25-hydroxyvitamin D and parathyroid hormone levels. J Bone Miner Res.

[24] Schleicher RL, Sternberg MR, Lacher DA (2016). The vitamin D status of the US population from 1988 to 2010 using standardized serum concentrations of 25-hydroxyvitamin D shows recent modest increases. Am J Clin Nutr.

[25] Belsky J, Sena S, Baqai S, Dilello LC, Petrini JR (2016). A four-year trend in serum 25-hydroxyvitamin D levels in western Connecticut. Conn Med.

[26] Khosravi-Boroujeni H, Sarrafzadegan N, Sadeghi M, Roohafza H, Ng SK, Pourmogaddas A, Ahmed F (2017). Prevalence and trends of vitamin D deficiency among Iranian adults: a longitudinal study from 2001–2013. J Nutr Sci Vitaminol (Tokyo).

[27] McKenna MJ, Murray BF, O’Keane M, Kilbane MT (2015). Rising trend in vitamin D status from 1993 to 2013: dual concerns for the future. Endocr Connect.

[28] Looker AC, Pfeiffer CM, Lacher DA, Schleicher RL, Picciano MF, Yetley EA (2008). Serum 25-hydroxyvitamin D status of the US population: 1988-1994 compared with 2000-2004. Am J Clin Nutr.

[29] Mirfakhraee S, Ayers CR, McGuire DK, Maalouf NM (2017). Longitudinal changes in serum 25-hydroxyvitamin D in the Dallas Heart Study. Clin Endocrinol (Oxf).

[30] Nielsen NO, Jørgensen ME, Friis H, Melbye M, Soborg B, Jeppesen C, Lundqvist M, Cohen A, Hougaard DM, Bjerregaard P (2014). Decrease in vitamin D status in the Greenlandic adult population from 1987–2010. PLoS ONE.

[31] Park JH, Hong IY, Chung JW, Choi HS (2018). Vitamin D status in South Korean population: seven-year trend from the KNHANES. Medicine (Baltimore).

[32] Sveriges meteorologiska och hydrologiska institut (Swedish Meteorological and Hydrological Institute) (2018) Års-och månadsstatistik (Yearly and monthly statistics). www.smhi.se/data/meteorologi/temperatur/2.1240. Accessed 9 Dec 2018

[33] Official Journal of the European Union (2006) Regulation no. 1925/2006 of the European parliament and of the council of 20 December 2006 on the addition of vitamins and minerals and of certain other substances to foods. www.eur-lex.europa.eu/legal-content/EN/TXT/HTML/?uri=CELEX:32006R1925&from=SV. Accessed 10 Dec 2018

[34] Livsmedelsverket (National Food Agency, Sweden) (2007) LIVSFS 2007:9 Föreskrifter om ändring i Livsmedelsverkets föreskrifter (SLVFS 1983:2) om berikning av vissa livsmedel (Regulations of changes in the National Food Agency’s regulations on the fortification of certain foods). www.livsmedelsverket.se/globalassets/om-oss/lagstiftning/berikn—kosttillsk—livsm-spec-gr-fsmp/livsfs-2007-9.pdf. Accessed 5 Dec 2018

[35] Holick MF, Binkley NC, Bischoff-Ferrari HA, Gordon CM, Hanley DA, Heaney RP, Murad MH, Weaver CM; Endocrine Society (2011). Evaluation, treatment, and prevention of vitamin D deficiency: an Endocrine Society clinical practice guideline. J Clin Endocrinol Metab.

[36] Wielders JP, Wijnberg FA (2009). Preanalytic stability of 25(OH)-vitamin D3 in human blood or serum at room temperature: solid as a rock. Clin Chem.

[37] Agborsangaya C, Toriola AT, Grankvist K (2010). The effects of storage time and sampling season on the stability of serum 25-hydroxy vitamin D and androstenedione. Nutr Cancer.

[38] Antoniucci DM, Black DM, Sellmeyer DE (2005). Serum 25-hydroxyvitamin D is unaffected by multiple freeze-thaw cycles. Clin Chem.

[39] Food and Nutrition Board, Institute of Medicine (2011). Dietary reference intakes for calcium and vitamin D.

