# Supplementary material for: Time trends of vitamin D concentrations in northern Sweden between 1986 and 2014: a population-based cross-sectional study
Source: Eur J Nutr. 2019 Nov 21;59(7):3037–44. doi: 10.1007/s00394-019-02142-x (PMC7501112; doi:10.1007/s00394-019-02142-x)
Supplement: Supplementary file 1 — Supplementary material 1 (DOCX 45 kb) [file 394_2019_2142_MOESM1_ESM.docx]

**Time trends of vitamin D concentrations in northern Sweden between 1986 and 2014**: **a population-based cross-sectional study**

**Journal name:** European Journal of Nutrition

**Authors:** Eva Summerhays^1^ **∙** Mats Eliasson^1^ **∙** Robert Lundqvist^2^ **∙** Stefan Söderberg^3^ **∙** Tanja Zeller^4,5^ **∙** Viktor Oskarsson^1^

^1^Sunderby Research Unit, Department of Public Health and Clinical Medicine, Umeå University, Umeå, Sweden

^2^Research and Innovation Unit, Norrbotten County Council, Luleå, Sweden

^3^Unit of Medicine, Department of Public Health and Clinical Medicine, Umeå University, Umeå, Sweden

^4^Department of General and Interventional Cardiology, University Heart Center Hamburg, Hamburg, Germany

^5^German Center for Cardiovascular Research, Partner Site Hamburg/Kiel/Luebeck, Hamburg, Germany

**Corresponding author:** Viktor Oskarsson (e-mail: viktor.oskarsson @umu.se)

**Online Resource 1** Vitamin D food-fortification policy in Sweden (as of May 2018)^a^

| Food item | Old policy | Current policy |
| --- | --- | --- |
| Milk (<1.5% fat)^b^ | 3.8–5.0 μg/L | 10 μg/L |
| Milk (1.5–3% fat) | Not obligatory | 10 μg/L |
| Cultured milk and yoghurt (<3% fat) | Not obligatory | 10 μg/L |
| Lactose-free items (<3% fat) | Not obligatory | 10 μg/L |
| Oat milk, soy milk, and rice milk | Not obligatory | 10 μg/L |
| Margarine and cooking fats^b^ | 7.5–10.0 μg/100 g | 20 μg/100 g |
| Liquid margarine and cooking fats | Not obligatory | 20 μg/100 g |

^a^Adopted from: https://www.livsmedelsverket.se/globalassets/publikationsdatabas/rapporter/2018/2018-nr-21-rad-om-d-vitamintillskott-till-riskgrupper.pdf

^b^In 2007, after regulations in the European Parliament and the European Council, fortification with vitamin D became obligatory for these food items (before which it was voluntary for non-organic items and prohibited for organic items)

**Online Resource 2** Bland-Altman plot of vitamin D concentrations measured by Abbott Architect and high-performance liquid chromatography (HPLC) in the study population in 2009 (*n* = 1522). The short dashed line represents the mean of the absolute value of the between-method difference (Abbott Architect minus HPLC), which was −8.4 ng/mL, whereas the long dashed lines represent 2 standard deviations of that mean (9.6)

**Online Resource 3** Vitamin D concentrations in the study population in 2009 by measurement method (Abbott Architect and HPLC) and according to sex and age

|  | Mean (SD) vitamin D concentration (ng/mL) | |  |
| --- | --- | --- | --- |
|  |  |  |  |
|  | Abbott Architect | HPLC | Mean (SD) difference^a^ |
| Overall population (*n* = 1522)^b^ | 19.6 (8.4) | 28.0 (9.0) | -8.4 (4.8) |
| Men *(n* = 764) | 19.2 (8.6) | 26.9 (8.6) | -7.7 (4.6) |
| Women (*n* = 758) | 19.9 (8.1) | 29.1 (9.2) | -9.1 (4.9) |
| Age <35 years (*n* = 229) | 19.1 (8.7) | 26.8 (9.6) | -7.7 (4.6) |
| Age 35–44 years (*n* = 306) | 18.2 (9.0) | 26.1 (9.8) | -7.9 (4.5) |
| Age 45–54 years *(n* = 303) | 18.8 (6.9) | 27.4 (7.5) | -8.7 (4.5) |
| Age ≥55 years (*n* = 684) | 20.8 (8.4) | 29.5 (8.8) | -8.7 (5.1) |

*HPLC* High-performance liquid chromatography, *SD* standard deviation

^a^Mean (SD) of the absolute value of the difference between the Abbott Architect method and the HPLC method

^b^The number of participants is different from that in the analytical cohort (*n* = 1554), because 32 participants with Abbott Architect data had no HPLC data

**Online Resource 4** Participant flow chart of the Northern Sweden MONICA study

12,130 participants eligible for analysis, 1986–2014

Invalid data (<8 or >160 ng/mL) on vitamin D (*n* = 373)

11,129 participants included for analysis, 1986–2014

Missing data on survey year (*n* = 33)

Missing data on vitamin D (*n* = 595)
